# Editorial: Subjective well-being and human decision behaviors

**DOI:** 10.3389/fpsyg.2025.1722414

**Published:** 2025-11-12

**Authors:** Thomas J. Huggins, Don J. Chai, Shibiao Wan, Chun Fong Lei

**Affiliations:** 1 School of Psychology, University of Monterrey, San Pedro Garza García, Mexico; 2 Beijing Normal – Hong Kong Baptist University, Zhuhai, China; 3 University of Nebraska Medical Center, Omaha, NE, United States; 4 Macau University of Science and Technology, Taipa, Macao SAR, China

**Keywords:** decision-making, wellbeing, neuroscience, consumers, education

The intersection of neuroscience, economics, and psychology has led to a tremendous growth in both scholarly and popular interest in decision-making research. The resulting field, of decision neuroscience, helps us understand the neural basis of decision-makings. Following the development of new technologies and methods, especially machine learning and AI, the complex relationships between brain activities and decision-making behaviors have become particularly promising.

Subjective wellbeing (SWB) is one, highly relevant perspective, that bridges individual emotions experienced within the brain with resulting human decision behaviors (Rutledge et al., 2015). Recent advances mean that the topic of SWB encompasses neurological and other biological data, including cutting edge psychometrics and a range of secondary data that can now be integrated through modern analytical methods. At the same time, data acquisition and analysis has been facilitated through artificial intelligence and machine learning. The current special topic has provided a way to position relevant studies, and to ensure that their results inform new approaches to important, real-world decision-making behaviors.

Our special topic includes research by Li et al., into the mediating effect of SWB on the positive relationship between perceived feedback and sense of belonging to school. Their analysis of data from 12,058 Chinese high school students indicates that SWB partially mediates this important relationship, as part of an indirect relationship between perceived feedback and sense of belonging to school (β = 0.47, *p* < 0.01). These results highlight the key role played by SWB in the pathway from educational dynamics to important life decisions being made by present day high school students.

An even larger-scale study, by Zhang and Duan, focused on the consumer behavior of software end-users. Their study contributed to the current special issue by introducing a hedonic element to models of end-user behavior. Extended free trials, of 7 instead of 3 days, resulted in an increase in paid use in the short term and in the years following the free trial period. In total, an extended free trial period resulted in a 20.93% increase in paid software usage, providing substantial support for the popular freemium model used by software developers and vendors.

Kondo et al. contributed their research into the impact of γ-aminobutyric acid (GABA) levels on decision making under risk. Their research applied a combination of functional magnetic resonance imaging and a behavioral protocol with 26 Japanese adults. Results indicated that higher levels of striatal GABA resulted in an increase in risk avoidance concerning a potential loss (r = −0.42, *p* = 0.038, 95% CI [−0.70, −0.03]). The results of this research indicate that frequent switching between brain states is associated with an increased sensitivity to loss and correspondingly aversive behaviors.

Huang and He's contribution to the special topic focused on life satisfaction among deaf or hard of hearing individuals. This study looked at the impacts of social media and found that participants used social media for communication, information and relaxation. Benefits of more social media use included higher levels of life satisfaction, perceived social support, and self-esteem; highlighting the potential for further research into the mediating effect of social support and self-esteem on the benefits of social media usage among deaf and hard of hearing individuals.

The special topic also includes research by Lu et al., who examined privacy concerns and disclosure behavior concerning short video platforms. Data from 302 TikTok platform users showed how privacy-related concerns mediate pathways between perceived risk, information sensitivity, and the effectiveness of privacy protection on disclosure behavior. This analysis provides a clear example of how SWB-related factors have a substantial impact on social media usage, in addition to more traditional predictors of platform user behavior.

As a whole, the current special topic has shown how dynamics related to SWB affect a wide range of decisions, from student commitment and retention, to paid software use and social media behavior, to loss averse decisions that may be accelerated through task and sensory switching. The studies included in this special issue have highlighted the potential for even more research into the relationship between elements of SWB and decision-making behaviors. The current collection of relevant research also highlights the value of integrating data, methods and models from fields as diverse as behavioral economics, neurology, and psychometric approaches to decision-making research.
